# Serum Osteoprotegerin Is Associated Independently with Peripheral Arterial Stiffness in Chronic Kidney Disease

**DOI:** 10.3390/diagnostics16121906

**Published:** 2026-06-19

**Authors:** Yahn-Bor Chern, Po-Yu Huang, Yu-Hsien Lai, Chih-Hsien Wang, Jen-Pi Tsai, Bang-Gee Hsu

**Affiliations:** 1 Division of Nephrology, Department of Medicine, Yuan’s General Hospital, Kaohsiung 802635, Taiwan; 2 Division of Nephrology, Department of Internal Medicine, Dalin Tzu Chi Hospital, Buddhist Tzu Chi Medical Foundation, Chiayi 62247, Taiwan; 3 Division of Nephrology, Hualien Tzu Chi Hospital, Buddhist Tzu Chi Medical Foundation, Hualien 97004, Taiwan; 4 Department of Pharmacology, School of Medicine, Tzu Chi University, Hualien 97004, Taiwan; 5 School of Medicine, Tzu Chi University, Hualien 97004, Taiwan

**Keywords:** peripheral arterial stiffness, brachial-ankle pulse wave velocity, chronic kidney disease, osteoprotegerin

## Abstract

**Background/Objectives**: Patients with chronic kidney disease (CKD) often present with peripheral arterial stiffness (PAS), which is associated with an increased cardiovascular risk. This study assessed the association between circulating osteoprotegerin (OPG), a known mediator of vascular calcification, and PAS, measured as brachial–ankle pulse wave velocity (baPWV), in patients with CKD. **Methods**: This cross-sectional investigation engaged 200 individuals with non-dialysis CKD. Serum OPG concentrations were measured using a commercial enzyme-linked immunosorbent assay. Participants were classified as having PAS when either left or right baPWV was greater than 18.0 m/s; those with baPWV values of 18.0 m/s or lower were assigned to the control group. **Results**: Eighty-six patients (43.0%) had PAS. In comparison to controls, PAS patients were older (*p* < 0.001) and had higher proportions of diabetes mellitus (*p* = 0.023) and hypertension (*p* = 0.010); systolic blood pressure was higher (*p* < 0.001), urine protein-to-creatinine ratio was elevated (*p* = 0.004), and serum OPG was markedly greater (*p* < 0.001), whereas estimated glomerular filtration rate was lower (*p* = 0.003). After full adjustment, OPG levels, in addition to older age and diabetes mellitus, demonstrated an independent association with PAS (odds ratio: 1.008; 95% confidence interval: 1.002–1.015; *p* = 0.010). The OPG level was positively associated with bilateral baPWV by Spearman’s correlation analysis (*p* < 0.001). **Conclusions**: Circulating OPG level showed an independent association with PAS and baPWV in CKD patients not yet on dialysis. Hence, OPG can be a potential marker of vascular risk in this patient population.

## 1. Introduction

Chronic kidney disease (CKD) remains a leading global health burden. A recent systematic review has reported that the worldwide age-standardized prevalence of CKD is 14.2% in 2023 [1]. As kidney function deteriorates in patients with CKD, vascular structure and function may undergo adverse remodeling, thereby increasing the likelihood of cardiovascular mortality [2,3]. Among the various clinical manifestations of vascular dysfunction related to this systemic pathology, arterial stiffness (AS), a vasculopathy characterized by diminished elasticity, has been considered a marker of both adverse cardiovascular outcomes and onset or progression of kidney disease [4,5,6].

Pulse wave velocity (PWV) is widely used in clinical settings as a noninvasive indicator of arterial stiffness and vascular compliance. In this study, peripheral arterial stiffness (PAS) in patients with CKD was evaluated using brachial–ankle PWV (baPWV). Compared with several traditional approaches for assessing central aortic stiffness, baPWV offers a simpler, more reproducible, and less operator-dependent method for estimating vascular condition. Accumulating evidence also indicates that baPWV has prognostic value for future cardiovascular events and mortality [7,8,9,10].

In CKD, disruption of the bone–vascular axis links disturbances in mineral metabolism to progressive vascular calcification, making it an important endogenous pathway contributing to arterial stiffness [11]. As a member of the tumor necrosis factor receptor superfamily, osteoprotegerin (OPG) is a key regulator of bone turnover and in the bone–vascular axis in CKD [12]. Produced by osteoblast-lineage cells, OPG acts as a soluble decoy receptor for receptor activator of nuclear factor kappa-B ligand (RANKL), inhibiting the differentiation, activation and bone resorption activity of osteoclasts [12]. Through this role, OPG participates in the regulation of bone turnover and mineral metabolism and has been implicated in the pathophysiology of CKD-mineral and bone disorder [11,12]. Circulating OPG levels are also increased in CKD, where they have been associated with vascular calcification and cardiovascular risk and mortality, emphasizing the strong coupling between bone injury and vascular injury in this population [13,14].

Although OPG has been implicated in CKD-related mineral bone disease, its association with PAS across the full spectrum of CKD stages remains poorly defined. Most previous studies have focused on dialysis cohorts or central arterial stiffness rather than peripheral arterial stiffness. Accordingly, whether OPG is consistently associated with PAS across the full spectrum of nondialysis CKD remains insufficiently defined. Therefore, the present study investigated the association between circulating OPG levels and PAS, assessed by baPWV, in individuals with nondialysis CKD. By validating this relationship, this study aimed to improve cardiovascular risk assessment and further evaluate the use of OPG as a potential marker of peripheral vascular health in CKD patients across various levels of kidney disease severity.

## 2. Materials and Methods

### 2.1. Research Design and Patient Enrollment

This study is cross-sectional in design, in which we recruited 200 patients with CKD from the nephrology department’s outpatient clinic at a tertiary referral center located in Hualien City, Taiwan, between January 2022 and December 2022. Power analysis determined that at least 113 patients were needed to detect a correlation coefficient of about 0.3 between serum OPG concentration and baPWV at a two-sided α of 0.05 with 90% statistical power [15]. CKD diagnosis was established in accordance with Kidney Disease: Improving Global Outcomes (KDIGO) guidelines, requiring two eGFR measurements obtained by the Chronic Kidney Disease Epidemiology Collaboration (CKD-EPI) equation at least 90 days apart. The exclusion criteria included patients with malignant disease, active infections within 3 months before enrollment, recent unstable angina or myocardial infarction, recent worsening of heart failure, cerebrovascular events, autoimmune conditions (including inflammatory bowel disease and rheumatoid arthritis), chronic obstructive pulmonary disease, end-stage renal disease or ongoing dialysis, and inability or refusal to provide informed consent. The study received ethical approval from the Research Ethics Committee of Hualien Tzu Chi Hospital, Buddhist Tzu Chi Medical Foundation (approval no. IRB108-219-A), and all participants provided written informed consent prior to enrollment. Information on patients’ demographic characteristics and biochemical variables was obtained from each participant’s electronic medical record. The data collected consisted of age, sex, CKD etiology, and use of antihypertensive or lipid-lowering medications. Resting blood pressure (BP) was recorded by a fixed examiner with a mercury-calibrated sphygmomanometer after 10 min of seated rest. Hypertension was defined as systolic BP (SBP) > 140 mmHg, diastolic BP (DBP) > 90 mmHg, or antihypertensive use within the preceding two weeks. Diabetes mellitus (DM) was diagnosed if fasting plasma glucose was ≥126 mg/dL, HbA1c was ≥6.5%, random glucose was ≥200 mg/dL on two separate occasions, or if the patient was already on antidiabetic treatment.

### 2.2. Collection of Anthropometric Data

Anthropometric measurements, including height and body weight, were recorded with participants barefoot and in light clothing, to the nearest 0.5 cm and 0.5 kg, respectively. Waist circumference was measured midway between the lowest rib margin and the iliac crest with a flexible tape. Body mass index (BMI) was then derived as weight (kg) divided by height squared (m^2^).

### 2.3. Assessment of Biochemical Data

Blood was drawn by venipuncture after an overnight fast, yielding approximately 5 mL per participant. A 0.5 mL aliquot was analyzed on a Sysmex XS-1000i hematology analyzer (Sysmex America, Mundelein, IL, USA) to determine white blood cell count and hemoglobin. Serum was isolated by centrifugation at 3000× *g* for 10 min. The resultant supernatant was then subjected to automated biochemical profiling on an Advia 1800 chemistry analyzer (Siemens Healthcare GmbH, Erlangen, Germany), encompassing fasting plasma glucose, total cholesterol (TCH), triglycerides (TG), low-density lipoprotein cholesterol (LDL-C), blood urea nitrogen (BUN), creatinine, albumin, total calcium, and phosphorus [16]. A random spot urine sample was collected, and the urine protein-to-creatinine ratio (UPCR) was measured. Intact parathyroid hormone (Abcam, Cambridge, MA, USA) and OPG concentrations (eBioscience Inc., San Diego, CA, USA) were each measured by enzyme-linked immunosorbent assay [17].

### 2.4. Detection of PAS Using baPWV

For baPWV, the pulse-wave propagation distance from the upper arm to the ankle was divided by the transit time between the corresponding waveform feet. All baPWV measurements were taken by a properly trained staff member using VP-2000 (Omron, Kyoto, Japan) [16]. We adopted a baPWV threshold of >18 m/s in either limb to define PAS, in line with the Physiological Diagnosis Criteria for Vascular Failure issued by the relevant Japanese expert committee [18]—a cutoff that reliably separates patients with elevated cardiovascular and hypertensive risk from those without. Any participant exceeding this threshold on either side was classified as PAS-positive.

### 2.5. Statistical Analysis

Prior to any group comparisons, all continuous variables were subjected to the Shapiro–Wilk test to assess distributional normality. Variables satisfying normality assumptions are described as mean ± standard deviation, with intergroup differences evaluated by the two-tailed independent-samples Student’s *t*-test. Non-normally distributed continuous variables are reported as median (interquartile range) and analyzed using the Mann–Whitney *U* test. Categorical data were expressed as counts with corresponding proportions and were compared across groups by Pearson’s chi-square test. Potential independent clinical factors associated with PAS were assessed by multivariable logistic regression, including all variables that had a *p* < 0.2 in the univariate analysis. Because TCH and LDL-C met this prespecified screening criterion, they were initially retained in the multivariable model as conventional cardiovascular covariates. Sensitivity analyses were further performed using alternative model specifications in which TCH and LDL-C were excluded, while smoking status and medication-related covariates, including ACEi/ARB and statin use, were additionally considered. Additional analyses were also performed with OPG standardized as a Z score to improve the clinical interpretability of the estimated odds ratio. Also, these models were constructed using regrouped CKD stage (stages 1–3 vs. stages 4–5) to further examine the robustness of the association with PAS. We used the variance inflation factor (VIF) to assess multicollinearity among candidate factors, with a VIF of <10 considered to demonstrate acceptable collinearity. A total of 1000 bootstrap resampling iterations were used to evaluate model robustness, and bias-corrected and accelerated (BCa) 95% confidence intervals (CI) were calculated. The serum OPG levels were compared across CKD stages using the one-way analysis of variance test. The associations between clinical variables and baPWV on either side, and OPG values, were assessed using Spearman’s correlation coefficients. Model calibration and clinical utility were evaluated using calibration plots. Receiver operating characteristic (ROC) curve analysis was performed to determine the optimal serum OPG cutoff value for identifying PAS in the CKD cohort, with the corresponding area under the curve (AUC) computed using MedCalc v22.019 (Ostend, Belgium). All statistical procedures were executed in SPSS v25.0 (IBM Corp., Armonk, NY, USA) and R v4.2.2 (R Foundation for Statistical Computing, Vienna, Austria). A two-tailed *p*-value < 0.05 was adopted as the threshold for statistical significance throughout all analyses.

## 3. Results

**Figure 1:**
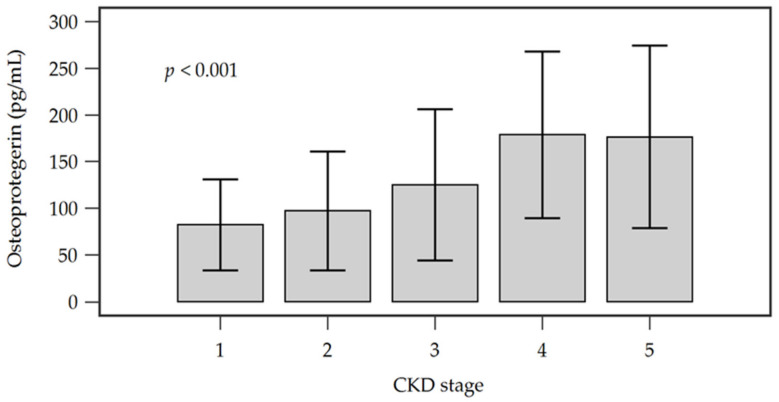
Comparison of serum osteoprotegerin levels across chronic kidney disease (CKD) stages.

The baseline clinical and biochemical characteristics of all 200 CKD patients are summarized in Table 1. Based on baPWV measurements, 86 (43.0%) patients were assigned to the PAS group and 114 (57.0%) to the control group. Compared with the reference group, PAS patients tended to be older (*p* < 0.001) and demonstrated higher SBP (*p* < 0.001), spot UPCR (*p* = 0.004), and serum OPG (*p* < 0.001), whereas their eGFR was significantly lower (*p* = 0.003). Additionally, DM and hypertension were more frequently encountered in the PAS group (*p* = 0.023 and *p* = 0.010, respectively). The distribution of CKD stages differed significantly between the two groups, with advanced stages more commonly observed in the PAS group (*p* = 0.010). As shown in Figure 1, patients with advanced CKD stages had higher circulating OPG levels, and the overall difference across the CKD stages was statistically significant (*p* < 0.001).

The multivariable logistic regression analysis revealed that OPG, age, and DM are independently associated with PAS (see Table 2). The results indicated odds ratios of 1.007 per 1 pg/mL increase in OPG (95% CI: 1.002–1.013, *p* = 0.011), 1.069 per additional year of age (95% CI: 1.027–1.112, *p* = 0.001), and 2.745 for the presence of DM (95% CI: 1.282–5.876, *p* = 0.009). In the final model, no significant associations were identified between hypertension, eGFR, spot UPCR, TCH, LDL-C, and creatinine with PAS. In sensitivity analyses using alternative model specifications, the independent association between OPG and PAS remained significant after excluding TCH and LDL-C and after additional adjustment for smoking status and medication-related covariates, including ACEi/ARB and statin use (Supplementary Table S1). When OPG was standardized as a Z score, each 1-standard deviation (SD) increase in OPG was independently associated with 1.91-fold higher odds of PAS (odds ratio, 1.907; 95% CI, 1.156–3.147; *p* = 0.012) (Supplementary Table S2). To address the potential influence of CKD stage, we performed an additional analysis using regrouped CKD stage (stages 1–3 vs. stages 4–5) instead of continuous renal function measures, and the association between OPG and PAS remained significant (Supplementary Table S3). In this additional model, ACEi/ARB use was inversely associated with PAS (odds ratio, 0.132; 95% CI, 0.019–0.916; *p* = 0.041) (Supplementary Table S3). All VIFs were <5, which indicated no concern for multicollinearity.

Bootstrap resampling further supported the robustness of these findings. OPG remained independently associated with PAS, and age and DM also remained independently related to PAS in the bootstrap-based model (Table 3).

**Figure 2:**
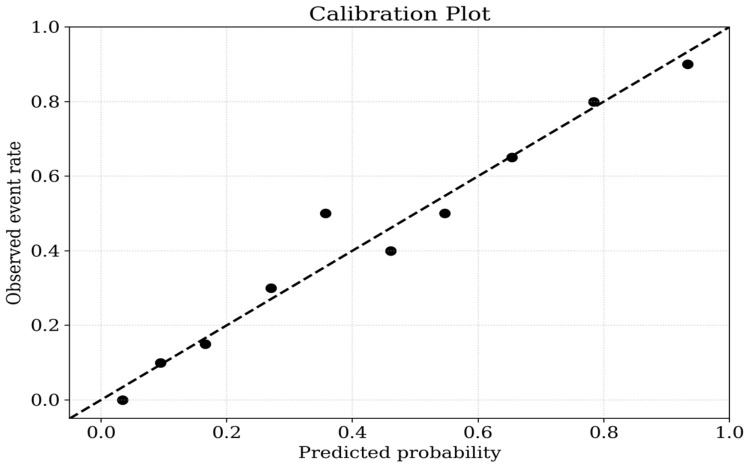
Calibration curve illustrating the agreement between predicted and observed probabilities of peripheral arterial stiffness by risk decile. The dashed diagonal line indicates ideal model calibration.

The overall fit and predictive performance of the OPG-based logistic regression model were further assessed. The Hosmer–Lemeshow test showed no significant difference between the observed and predicted risks (χ^2^ = 3.494, *p* = 0.900), indicating that the model’s probability estimates had excellent calibration and a high reliability. The calibration assessment showed a close agreement between the predicted and observed PAS risks across deciles of predicted probability (Figure 2). The model had excellent calibration. The calibration intercept was 0.000 (95% CI: −0.349 to 0.349), suggesting perfect calibration-in-the-large with no systematic overestimation or underestimation of the risk. Further, the calibration slope was exactly 1.000 (95% CI: 0.699–1.130), showing that the predictor effects were optimally estimated without overfitting. The overall probabilistic accuracy was good, as reflected by a Brier score of 0.165. Because lower Brier scores indicate better prediction, with 0 representing perfect accuracy, this value suggests acceptable overall probabilistic performance of the model in the present cohort.

**Figure 3:**
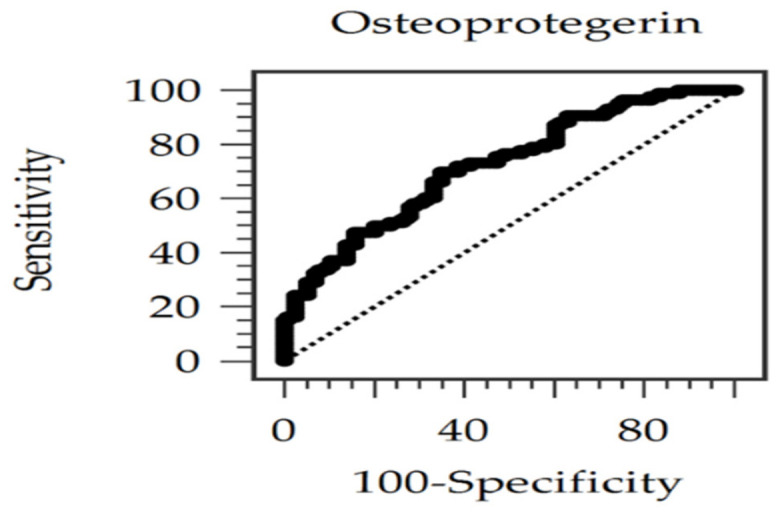
Receiver operating characteristic curve demonstrating the discriminatory capacity of serum osteoprotegerin for peripheral arterial stiffness among 200 individuals with chronic kidney disease.

ROC analysis results are shown in Figure 3. The AUC of 0.721 (95% CI 0.651–0.792; *p* < 0.0001) places OPG in the moderate discriminator range. At the optimal cutoff of 127.53 pg/mL, sensitivity was 69.77%, and specificity was 64.91%; the positive predictive value was 60%, and the negative predictive value was 74%.

Table 4 presents the correlation matrix for OPG and clinical variables. Higher OPG levels were related to greater arterial stiffness, as reflected by both left baPWV (ρ = 0.445, *p* < 0.001) and right baPWV (ρ = 0.427, *p* < 0.001). For baPWV, comparable findings were observed bilaterally. Both left and right baPWV increased with age, SBP, DBP, and UPCR, whereas lower eGFR was linked to higher baPWV values. Specifically, the correlation coefficients for left and right baPWV were as follows: age, ρ = 0.440 and 0.457; SBP, ρ = 0.434 and 0.418; DBP, ρ = 0.266 and 0.242; UPCR, ρ = 0.176 and 0.178; and eGFR, ρ = −0.208 and −0.188, respectively. All of these associations reached statistical significance. When OPG was examined in relation to other clinical measures, higher values were observed in association with older age (ρ = 0.459), higher SBP (ρ = 0.358), increased BUN (ρ = 0.277), greater creatinine (ρ = 0.321), and higher UPCR (ρ = 0.212). Conversely, OPG decreased with increasing albumin (ρ = −0.206), hemoglobin (ρ = −0.353), and eGFR (ρ = −0.426). These relationships were all statistically significant. In contrast, OPG did not show meaningful correlations with body mass index, DBP, total cholesterol, triglycerides, LDL-C, glucose, calcium, or phosphorus.

## 4. Discussion

In this study, multivariable logistic regression with bootstrap resampling showed that higher serum OPG levels, older age, and DM were independently associated with PAS in patients across the CKD spectrum. In addition, OPG showed moderate discriminatory ability for identifying PAS, with an AUC of 0.721. We also found that OPG was positively correlated with bilateral baPWV, SBP, BUN, creatinine, and UPCR, and inversely correlated with albumin, hemoglobin, and eGFR by Spearman’s correlation analysis. Previous human studies have also linked circulating OPG to central vascular abnormalities. Carotid–femoral pulse wave velocity (cfPWV) is largely regarded as the gold standard of preclinical assessment for aortic stiffness via noninvasive measurement [19]. Scialla et al. assessed serum OPG in 351 subjects, of which 226 had aortic PWV data available, and also found that compared to the lowest tertile of OPG, aortic PWV was about 10% greater in the highest OPG tertile after multivariable adjustment [20]. In a hemodialysis cohort of 120 patients, 53 patients (44.2%) had high central AS, defined as cfPWV greater than 10 m/s; increasing OPG tertiles independently predicted high central AS [21]. Similarly, in hypertensive patients, log-transformed OPG remained independently associated with cfPWV, with a regression coefficient of 1.736 (95% CI, 0.809–2.663; *p* < 0.001) [22]. In addition to PWV, another systematic review and meta-analysis including 7642 participants revealed that elevated OPG was related to higher odds of coronary artery calcification (pooled odds ratio, 1.15; 95% CI, 1.03–1.30) [23].

For the present study, we employed baPWV, which is an easier and more practical method for routine use in clinical practice as it requires only four-limb cuff placement and has less operator-dependent prevalence [7]. While baPWV is not the same as cfPWV, human studies have demonstrated a significant correlation between the two measurements. In 409 healthy adults, baPWV was strongly correlated with aortic PWV (*r* = 0.76), and the change in baPWV after exercise intervention was also positively correlated with the change in aortic PWV (*r* = 0.74) [24]. Therefore, our findings support the view that OPG is associated with arterial stiffening in CKD and extend the existing literature by showing a similar relationship with baPWV, a more convenient vascular assessment tool.

The current study adds strength to previous evidence showing that traditional cardiovascular risk factors contribute to PAS in the population with CKD. Aging is an established trigger of vascular dysregulation involving the structural reprogramming of the arteries, and age-related anthropometric alterations [25,26]. DM overlaps mechanistically with PAS through endothelial dysfunction and glycation-mediated vascular injury, both further potentiated in CKD [27,28]. Moreover, hypertension was significantly more prevalent in the PAS group, thereby further promoting arterial stiffening via mechanical strain and endothelial injury [29,30].

Impaired renal function is associated with vascular stiffness, and baPWV values were more likely to increase as eGFR decreases. This finding might be explained by chronic inflammation, hemodynamic alterations, oxidative stress, vascular calcification, and the accumulation of uremic toxins related to CKD [31,32,33]. UPCR was also significantly positively associated with baPWV. Consistent with this bidirectional association, proteinuria likely reflects extensive renal and endothelial injury. Meanwhile, stiffened arteries can further worsen renal microcirculatory impairment [34,35,36].

The pathogenic role of OPG in CKD is intimately associated with the bone–vascular axis. In addition to the well-established fact that OPG inhibits osteoclast activation via its interaction with RANKL, the serum OPG level is significantly higher in individuals with reduced kidney function [37]. Accordingly, this elevation is considered a compensatory but, ultimately, inadequate mechanism in response to chronic vasculopathy and systemic calcification processes [38]. The associations observed between OPG levels as well as SBP, BUN and creatinine levels, and UPCR indicate an interrelated pathophysiology, whereby OPG may be a marker of the myriad metabolic and vascular derangements that accompany CKD.

The association of OPG and AS might be complex, with both direct and indirect pathogenic links involved in their relationships. Experimental evidence suggests that OPG may function, at least in part, as a compensatory inhibitor of vascular mineralization. In animal models, OPG deficiency has been associated with medial calcification of the aorta and renal arteries, whereas exogenous OPG has been shown to reduce vascular calcification [39,40]. Furthermore, in vitro studies found that OPG can modulate vascular smooth muscle cell calcification, while endothelial studies indicated that OPG may also enhance inflammatory vascular injury under pro-inflammatory conditions by promoting adhesion molecule expression and monocyte binding [41,42]. Collectively, these findings suggest that elevated circulating OPG in CKD may reflect both disturbed bone–vascular signaling and a reactive response to calcific and inflammatory stress, which may help explain its association with PAS in our cohort.

More specifically, several pathophysiological mechanisms could potentially be responsible for the inverse association between OPG and albumin levels. In particular, the association might reflect an increased loss of albumin with proteinuria. Further, uremia-associated changes in albumin levels might compromise vascular health, thereby eliciting additional synthesis or secretion of OPG as a compensatory signal [43,44]. In a similar fashion, the inverse association between OPG and hemoglobin levels may be partly caused by the systemic inflammation observed in both CKD-related anemia and calcification processes [45]. However, these interpretations remain hypothesis-generating and should be viewed cautiously. Further studies are needed to validate these associations and clarify whether OPG directly contributes to vascular remodeling or primarily reflects ongoing vascular injury.

In the current study, serum OPG was independently associated with PAS in patients with non-dialysis CKD and provided moderate discrimination for prevalent PAS (AUC = 0.721). AS has been linked with cardiovascular risk in CKD across various studies [31], but relative to the existing KDIGO framework for risk assessment, much of this information should remain based in clinical practice on the cause of kidney disease, glomerular filtration rate, and albuminuria, as well as validated cardiovascular risk tools [3]. In this setting, OPG may be more appropriately regarded as a supplementary biomarker rather than an alternative risk assessment tool. The current findings also suggest that OPG may provide additional information beyond standard clinical variables such as age, blood pressure, and diabetes status, although its clinical value should still be regarded as supportive rather than definitive. In our further analyses, the OPG-PAS association remained significant under alternative model parameterizations, including excluding lipid variables, controlling for smoking and medication use, and substituting continuous measures of kidney function with CKD stages as categorical variables. When expressed on a common scale, each 1-SD-unit increment of OPG associates with nearly 1.9 times the odds of PAS, which is clinically more interpretable than the odds ratio per pg/mL. Concerning the univariate analysis, which did not identify a significant relation with the use of renin–angiotensin–aldosterone system blockers, this became statistically significant after multivariable adjustment, probably meaning that there is confounding by indication. This is due to the fact that ACEi/ARB therapy is provided for hypertensive, albuminuric, or more advanced CKD patients who are also at higher odds for AS. After adjustment for these interrelated factors, the inverse association between ARB use and PAS became apparent. Nevertheless, this finding should be interpreted with caution, given the relatively small sample size and wide CI. OPG may therefore help identify patients with a greater vascular stiffness burden who may merit closer cardiovascular evaluation; however, future studies are still required to determine whether adding OPG to existing CKD risk assessment strategies would provide incremental clinical benefit beyond established tools. However, because the observed AUC was only moderate and the cutoff value of 127.53 pg/mL was derived from the present cohort, the current findings do not support routine OPG testing for PAS risk assessment or direct generalization of this threshold to other CKD populations without prospective validation, external confirmation, and clear evidence of added clinical utility beyond existing tools [3,13,14]. This investigation is constrained by several limitations. First, because the study used a cross-sectional design, it was not possible to establish whether OPG levels contribute causally to PAS severity. Thus, longitudinal studies should be conducted to define the stage-specific effects of OPG. Second, despite adjusting for important covariates, residual confounding from the complex pathways of metabolic and inflammatory derangements that drive CKD-related vascular disturbances could not be ruled out. Third, this study assessed only total circulating OPG concentrations and did not include RANKL, the OPG/RANKL ratio, other CKD-mineral and bone disorder parameters, inflammatory biomarkers, or representative uremic toxins, which limited a more comprehensive mechanistic interpretation of the observed association between OPG and PAS. Future work incorporating these biomarkers may provide a clearer understanding of whether elevated OPG primarily reflects disturbances in bone turnover, vascular injury, or both. Fourth, although we performed an additional multivariable analysis using regrouped CKD stage, the stage grouping itself was not significantly associated with PAS. Therefore, we did not proceed with further stage-stratified correlation analyses, and this approach still does not replace a formal subgroup analysis across CKD severity. Fifth, caution is needed when applying these findings to broader patient groups, as the study cohort was drawn from a single Taiwanese medical center. Thus, intervention trials examining approaches that regulate the OPG/RANKL pathway for cardiovascular protection are highly recommended.

## 5. Conclusions

Serum OPG levels demonstrated an independent association with PAS in patients with CKD who are not receiving dialysis. Elevated OPG values exhibited a statistically significant relationship with PAS and were positively correlated with baPWV. Based on these findings, OPG can be an innovative biomarker of peripheral vascular dysfunction and may improve cardiovascular risk assessment throughout the course of CKD.

## Figures and Tables

**Table 1:** Clinical characteristics of the 200 subjects identified with chronic kidney disease, classified according to the existence or non-existence of peripheral arterial stiffness.

Features	All Patients (*n* = 200)	Control Group (*n* = 114)	PAS Group (*n* = 86)	*p* Value
Age (years)	67.86 ± 13.92	63.21 ± 13.72	74.01 ± 11.68	<0.001 *
Height (cm)	159.01 ± 7.81	159.88 ± 7.21	157.85 ± 8.44	0.069
Body weight (kg)	65.45 ± 12.24	66.37 ± 13.05	64.27 ± 11.03	0.230
Body mass index (kg/m^2^)	25.80 ± 3.93	25.87 ± 4.28	25.71 ± 3.44	0.776
Left baPWV (m/s)	17.06 ± 3.21	14.85 ± 1.72	19.99 ± 2.22	<0.001 *
Right baPWV (m/s)	17.04 ± 3.16	14.87 ± 1.86	19.92 ± 2.03	<0.001 *
SBP (mmHg)	139.12 ± 23.84	132.61 ± 21.50	147.76 ± 24.15	<0.001 *
DBP (mmHg)	78.34 ± 12.86	72.01 ± 11.44	75.24 ± 14.39	0.078
Total cholesterol (mg/dL)	154.00 (139.00–177.00)	150.50 (136.00–173.00)	161.50 (140.75–182.25)	0.114
Triglyceride (mg/dL)	121.50 (89.00–165.00)	119.50 (88.75–163.00)	125.00 (88.75–171.50)	0.843
LDL-C (mg/dL)	88.39 ± 31.93	85.33 ± 32.93	92.44 ± 30.27	0.119
Pre-prandial glucose (mg/dL)	106.50 (95.00–136.75)	103.00 (94.00–132.25)	108.50 (96.00–139.50)	0.263
Blood urea nitrogen (mg/dL)	30.50 (22.00–48.00)	28.50 (20.00–50.25)	33.50 (23.75–46.00)	0.287
Creatinine (mg/dL)	1.75 (1.30–2.78)	1.60 (1.20–2.73)	1.85 (1.40–2.83)	0.121
eGFR (mL/min)	37.46 ± 23.42	41.75 ± 25.86	31.78 ± 18.39	0.003 *
Spot UPCR (g/g)	0.55 (0.26–1.19)	0.44 (0.22–1.06)	0.68 (0.35–1.55)	0.004 *
Albumin (g/dL)	4.00 (3.80–4.20)	4.00 (3.80–4.20)	4.00 (3.80–4.20)	0.284
Hemoglobin (g/dL)	11.75 ± 2.05	11.89 ± 2.11	11.57 ± 1.97	0.279
Total calcium (mg/dL)	8.97 (8.78–9.24)	8.94 (8.81–9.24)	9.00 (8.76–9.32)	0.844
Phosphorus (mg/dL)	3.70 (3.50–4.20)	3.70 (3.50–4.20)	3.70 (3.40–4.20)	0.545
Osteoprotegerin (pg/mL)	128.14 (82.33–175.80)	107.98 (70.01–149.88)	154.30 (110.89–230.94)	<0.001 *
Female, *n* (%)	87 (43.5)	46 (40.4)	41 (47.7)	0.301
Diabetes mellitus, *n* (%)	84 (42.0)	40 (35.1)	44 (51.2)	0.023 *
Hypertension, *n* (%)	119 (59.5)	59 (51.8)	60 (69.8)	0.010 *
Glomerulonephritis, *n* (%)	41 (20.5)	22 (19.3)	19 (22.1)	0.628
ARB use, *n* (%)	109 (54.5)	58 (50.9)	51 (59.3)	0.236
β-blocker use, *n* (%)	57 (28.5)	32 (28.1)	25 (29.1)	0.877
CCB use, *n* (%)	72 (36.0)	39 (34.2)	33 (38.4)	0.544
α-adrenergic blocker use, *n* (%)	34 (17.0)	18 (15.8)	16 (18.6)	0.600
Statin use, *n* (%)	92 (46.0)	53 (46.5)	39 (45.3)	0.873
Fibrate use, *n* (%)	39 (19.5)	23 (20.2)	16 (18.6)	0.781
CKD stage 1, *n* (%)	10 (5.0)	10 (8.8)	0 (0)	0.010 *
CKD stage 2, *n* (%)	20 (10.0)	13 (11.4)	7 (8.1)	
CKD stage 3, *n* (%)	81 (40.5)	49 (43.0)	32 (37.2)	
CKD stage 4, *n* (%)	50 (25.0)	21 (18.4)	29 (33.7)	
CKD stage 5, *n* (%)	39 (19.5)	21 (18.4)	18 (21.0)	

Data are presented as mean ± standard deviation, median (interquartile range), or *n* (%), as appropriate. Abbreviations: SBP, systolic blood pressure; DBP, diastolic blood pressure; LDL-C, low-density lipoprotein cholesterol; eGFR, estimated glomerular filtration rate; UPCR, urine protein-to-creatinine ratio; CKD, chronic kidney disease; PAS, peripheral arterial stiffness; baPWV, brachial–ankle pulse wave velocity; ARB, angiotensin receptor blocker; CCB, calcium channel blocker. * *p* < 0.05.

**Table 2:** Independent factors associated with peripheral arterial stiffness among 200 chronic kidney disease patients, assessed using multivariable logistic regression analysis.

Clinical Parameters	Odds Ratio	95% CI	*p* Value
Osteoprotegerin, 1 pg/mL	1.007	1.002—1.013	0.011 *
Age, 1 year	1.069	1.027—1.112	0.001 *
Diabetes mellitus, present	2.745	1.282—5.876	0.009 *
Hypertension, present	0.900	0.316—2.566	0.844
Systolic blood pressure, 1 mmHg	1.012	0.980—1.045	0.468
Diastolic blood pressure, 1 mmHg	1.029	0.980—1.080	0.255
Total cholesterol, 1 mg/dL	0.994	0.978—1.012	0.522
Low-density lipoprotein cholesterol, 1 mg/dL	1.013	0.994—1.033	0.192
eGFR, 1 mL/min	0.979	0.951—1.008	0.155
Creatinine, 1 mg/dL	0.732	0.485—1.104	0.137
Spot UPCR, 1 g/g	1.261	0.909—1.749	0.164

Odds ratios are presented per unit increase or category, as indicated in the first column. The multivariable model included clinically relevant covariates selected from the baseline analysis. Additional sensitivity and alternative model analyses are provided in Supplementary Tables S1–S3. Abbreviations: CI, confidence interval; eGFR, estimated glomerular filtration rate; LDL-C, low-density lipoprotein cholesterol; UPCR, urine protein-to-creatinine ratio; PAS, peripheral arterial stiffness; CKD, chronic kidney disease. * *p* < 0.05.

**Table 3:** Multivariable logistic regression analysis incorporating bootstrap resampling (B = 1000) to evaluate factors independently associated with peripheral arterial stiffness among 200 chronic kidney disease participants.

Clinical Parameters	B	BCa 95% CI	*p* Value
Osteoprotegerin, per 1 pg/mL	0.007	0.001, 0.018	0.005
Age, per 1 year increase	0.067	0.025, 0.138	0.002
Diabetes mellitus	1.010	0.118, 2.040	0.005
Hypertension	−0.105	−1.401, −1.114	0.834
Systolic blood pressure, per 1 mmHg	0.012	−0.028, 0.055	0.509
Diastolic blood pressure, per 1 mmHg	0.028	−0.028, 0.099	0.271
Total cholesterol, per 1 mg/dL	−0.006	−0.027, 0.012	0.532
LDL-C, per 1 mg/dL	0.013	−0.012, 0.053	0.221
eGFR, per 1 mL/min increase	−0.021	−0.050, −0.001	0.148
Creatinine, per 1 mg/dL	−0.312	−0.777, −0.042	0.157
Spot UPCR, per 1 g/g increase	0.232	−0.272, 1.296	0.268

Regression coefficients (B) represent log-odds estimates. BCa denotes bias-corrected and accelerated; CI, confidence interval; LDL-C, low-density lipoprotein cholesterol.

**Table 4:** Spearman correlation matrix showing the relationships of bilateral baPWV and log-transformed osteoprotegerin with clinical and biochemical parameters in 200 individuals with chronic kidney disease.

Parameters	Left-Side baPWV (m/s)		Right-Side baPWV (m/s)		OPG (pg/mL)	
	Spearman’s Rho	*p* Value	Spearman’s Rho	*p* Value	Spearman’s Rho	*p* Value
Age (years)	0.440	<0.001 *	0.457	<0.001 *	0.459	<0.001 *
BMI (kg/m^2^)	0.139	0.050	0.128	0.070	−0.012	0.864
Left baPWV (m/s)	—	—	0.930	<0.001 *	0.445	<0.001 *
Right baPWV (m/s)	0.930	<0.001 *	—	—	0.427	<0.001 *
OPG (pg/mL)	0.445	<0.001 *	0.427	<0.001 *	—	—
SBP (mmHg)	0.434	<0.001 *	0.418	<0.001 *	0.358	<0.001 *
DBP (mmHg)	0.266	<0.001 *	0.242	0.001 *	0.121	0.087
TCH (mg/dL)	0.099	0.162	0.113	0.113	0.034	0.633
Triglyceride (mg/dL)	0.024	0.739	0.064	0.365	−0.010	0.885
LDL-C (mg/dL)	0.108	0.129	0.136	0.055	−0.002	0.981
Glucose (mg/dL)	0.046	0.522	0.052	0.468	0.059	0.408
Albumin (g/dL)	0.001	0.996	0.006	0.935	−0.206	0.003 *
Hemoglobin (g/dL)	−0.004	0.950	−0.019	0.795	−0.353	<0.001 *
BUN (mg/dL)	0.054	0.450	0.044	0.538	0.277	<0.001 *
Creatinine (mg/dL)	0.078	0.270	0.056	0.431	0.321	<0.001 *
eGFR (mL/min)	−0.208	0.003 *	−0.188	0.008 *	−0.426	<0.001 *
Calcium (mg/dL)	0.056	0.430	0.043	0.548	−0.052	0.463
Phosphorus (mg/dL)	−0.010	0.884	−0.021	0.767	0.110	0.119
UPCR (g/g)	0.176	0.013 *	0.178	0.012 *	0.212	0.003 *

Correlations were examined using Spearman’s rank correlation test. Abbreviations: OPG, osteoprotegerin; BMI, body mass index; baPWV, brachial–ankle pulse wave velocity; SBP, systolic blood pressure; DBP, diastolic blood pressure; TCH, total cholesterol; LDL-C, low-density lipoprotein cholesterol; BUN, blood urea nitrogen; eGFR, estimated glomerular filtration rate; UPCR, urine protein-to-creatinine ratio. The symbol “—” indicates “not applicable.” * *p* < 0.05.

## Data Availability

The datasets generated and/or analyzed in the present study are available from the corresponding author upon reasonable request.

## Supplementary Materials

The following supporting information can be downloaded at: https://www.mdpi.com/article/10.3390/diagnostics16121906/s1; Table S1: Sensitivity analysis of the association between serum osteoprotegerin and peripheral arterial stiffness after exclusion of total cholesterol and low-density lipoprotein cholesterol and additional adjustment for smoking and medication-related covariates. Table S2: Multivariable logistic regression analysis using standardized osteoprotegerin for peripheral arterial stiffness. Table S3: Additional multivariable logistic regression analysis using standardized osteoprotegerin, regrouped chronic kidney disease stage, and ACEi/ARB adjustment for peripheral arterial stiffness.

## Abbreviations

The following abbreviations are used in this manuscript:

AS	Arterial stiffness
AUC	Area under the receiver operating characteristic curve
baPWV	Brachial–ankle pulse wave velocity
BMI	Body mass index
BP	Blood pressure
BUN	Blood urea nitrogen
CKD	Chronic kidney disease
CKD-EPI	Chronic Kidney Disease Epidemiology Collaboration
CI	Confidence interval
CVD	Cardiovascular disease
DBP	Diastolic blood pressure
DM	Diabetes mellitus
eGFR	Estimated glomerular filtration rate
KDIGO	Kidney Disease Improving Global Outcomes
LDL-C	Low-density lipoprotein cholesterol
OPG	Osteoprotegerin
RANKL	Receptor activator of nuclear factor kappa-B ligand
PAS	Peripheral arterial stiffness
PWV	Pulse wave velocity
ROC	Receiver operating characteristic curve
SBP	Systolic blood pressure
SD	Standard deviation
TCH	Total cholesterol
TG	Triglyceride
TRAIL	TNF-related apoptosis-inducing ligand
UPCR	Urine protein-to-creatinine ratio
