# Supplementary material for: Serum Osteoprotegerin Is Associated Independently with Peripheral Arterial Stiffness in Chronic Kidney Disease
Source: Diagnostics (Basel). 2026 Jun 19;16(12):1906. doi: 10.3390/diagnostics16121906 (PMC13297961; doi:10.3390/diagnostics16121906)
Supplement: Supplementary file 1 [file diagnostics-16-01906-s001.zip › diagnostics-4292768-supplementary.pdf]

Supplementary Table S1. Sensitivity analysis of the association between serum osteoprotegerin and peripheral arterial stiffness after exclusion of total cholesterol and low-density lipoprotein cholesterol and additional adjustment for smoking and medication-related covariates.

Variable	Odds ratio	95% CI	*p* Value
Osteoprotegerin	1.007	1.001 to 1.013	0.016*
Age	1.076	1.032 to 1.121	0.001*
DM	2.704	1.230 to 5.944	0.013
Hypertension	6.955	0.887 to 54.530	0.065
SBP	1.009	0.976 to 1.043	0.605
DBP	1.036	0.987 to 1.088	0.154
eGFR	0.974	0.946 to 1.004	0.089
Cre	0.693	0.460 to 1.044	0.080
UPCR	1.304	0.928 to 1.833	0.126
Smoking	0.734	0.221 to 2.436	0.613
ACEi/ARB	0.111	0.016 to 0.783	0.027
Statin	1.649	0.758 to 3.587	0.207

Sensitivity analyses were performed using an alternative multivariable logistic regression model derived from the primary model after exclusion of total cholesterol and LDL-C and additional inclusion of smoking status, ACEi/ARB use, and statin use. Abbreviations: CI, confidence interval; DM, diabetes mellitus; SBP, systolic blood pressure; DBP, diastolic blood pressure; eGFR, estimated glomerular filtration rate; Cre, creatinine; UPCR, urine protein-to-creatinine ratio; ACEi, angiotensin-converting enzyme inhibitor; ARB, angiotensin receptor blocker. * $p < 0.05$.

Supplementary Table S2. Multivariable logistic regression analysis using standardized osteoprotegerin for peripheral arterial stiffness.

Variable	Odds ratio	95% CI	*p* Value
OPG, per 1-SD increase	1.907	1.156 to 3.147	0.012*
Age, 1 year	1.069	1.027 to 1.112	0.001*
Diabetes mellitus, present	2.748	1.282 to 5.876	0.009*
Hypertension, present	0.900	0.316 to 2.566	0.844
SBP, 1 mmHg	1.012	0.980 to 1.045	0.468
DBP, 1 mmHg	1.029	0.980 to 1.080	0.255
Cholesterol, 1 mg/dL	0.994	0.978 to 1.012	0.522
LDL-C, 1 mg/dL	1.013	0.994 to 1.033	0.192

eGFR, 1 mL/min/1.73 m^2	0.979	0.951 to 1.008	0.155
Creatinine, 1 mg/dL	0.732	0.485 to 1.105	0.137
UPCR, 1 g/g	1.261	0.909 to 1.749	0.164

OPG was standardized as a Z score to improve the clinical interpretability of the estimated effect size. Abbreviations: CI, confidence interval; OPG, osteoprotegerin; SD, standard deviation; SBP, systolic blood pressure; DBP, diastolic blood pressure; LDL-C, low-density lipoprotein cholesterol; eGFR, estimated glomerular filtration rate; UPCR, urine protein-to-creatinine ratio. * $p < 0.05$.

Supplementary Table S3. Additional multivariable logistic regression analysis using standardized osteoprotegerin, regrouped chronic kidney disease stage, and ACEi/ARB adjustment for peripheral arterial stiffness.

Variable	Odds ratio	95% CI	*p* Value
OPG, per 1-SD increase	1.006	1.001 to 1.012	0.024*
Age, 1 year	1.085	1.043 to 1.128	<0.001*
Diabetes mellitus, present	3.100	1.434 to 6.699	0.004*
Hypertension, present	5.681	0.755 to 42.733	0.092
SBP, 1 mmHg	1.011	0.979 to 1.044	0.502
DBP, 1 mmHg	1.034	0.986 to 1.085	0.170
Regrouped CKD stages (CKD stages 4–5 vs. stages 1–3)	1.419	0.667 to 3.015	0.363
UPCR, 1 g/g	1.213	0.900 to 1.635	0.205
ACEi/ARB use, yes	0.132	0.019 to 0.916	0.041*

In this additional model, OPG was standardized as a Z score to improve clinical interpretability, and CKD severity was represented by regrouped CKD stage (stages 1–3 vs. stages 4–5) rather than continuous renal function measures. ACEi/ARB adjustment was additionally considered in the model. Abbreviations: CI, confidence interval; OPG, osteoprotegerin; SD, standard deviation; CKD, chronic kidney disease; SBP, systolic blood pressure; DBP, diastolic blood pressure; UPCR, urine protein-to-creatinine ratio; ACEi, angiotensin-converting enzyme inhibitor; ARB, angiotensin receptor blocker. * $p < 0.05$.
